# The air-sea interface and surface stress under tropical cyclones

**DOI:** 10.1038/srep05306

**Published:** 2014-06-16

**Authors:** Alexander V. Soloviev, Roger Lukas, Mark A. Donelan, Brian K. Haus, Isaac Ginis

**Affiliations:** 1 Oceanographic Center, Nova Southeastern University, Dania Beach, Florida; 2 Rosenstiel School of Marine and Atmospheric Science, University of Miami, Florida; 3 Department of Oceanography, University of Hawaii at Manoa, Honolulu, Hawaii; 4 Graduate School of Oceanography, University of Rhode Island, Narragansett, Rhode Island

## Abstract

Tropical cyclone track prediction is steadily improving, while storm intensity prediction has seen little progress in the last quarter century. Important physics are not yet well understood and implemented in tropical cyclone forecast models. Missing and unresolved physics, especially at the air-sea interface, are among the factors limiting storm predictions. In a laboratory experiment and coordinated numerical simulation, conducted in this work, the microstructure of the air-water interface under hurricane force wind resembled Kelvin-Helmholtz shear instability between fluids with a large density difference. Supported by these observations, we bring forth the concept that the resulting two-phase environment suppresses short gravity-capillary waves and alters the aerodynamic properties of the sea surface. The unified wave-form and two-phase parameterization model shows the well-known increase of the drag coefficient (*C_d_*) with wind speed, up to ~30 ms^−1^. Around 60 ms^−1^, the new parameterization predicts a local peak of *C_k_/C_d_*, under constant enthalpy exchange coefficient *C_k_*. This peak may explain rapid intensification of some storms to major tropical cyclones and the previously reported local peak of lifetime maximum intensity (bimodal distribution) in the best-track records. The bimodal distribution of maximum lifetime intensity, however, can also be explained by environmental parameters of tropical cyclones alone.

The primary elements contributing to numerical tropical cyclone forecast success (within the intrinsic predictability timescale limits^1^,^2^) are physics, computational power, and observations. Discretized physics are essential for numerical models of tropical cyclones, along with parameterization of processes occurring on unresolved spatial and temporal scales. Computational performance is important for improved numerical grid resolution, more sophisticated physics and for timely operational data assimilation and multi-ensemble forecasting. Observations contribute to specification of the initial vortex and the evolving ocean-atmosphere environment, and are essential for testing predictions. During the last quarter-century, computational power increased by orders of magnitude; in addition, more extensive and intensive tropical cyclone observations are now made. Nevertheless, storm intensity prediction, including the problem of rapid storm intensification, has seen little progress^3^,^4^. Substantial improvement in computations and observations suggests that poorly parameterized or missing physics are the weakest component in tropical cyclone prediction systems.

Tropical cyclones take heat energy from the ocean and dissipate kinetic energy in the ocean via the air-sea interface. The theoretical maximum intensity *V* that a steady state tropical cyclone can attain, or potential intensity^5^,^6^, is given by equation 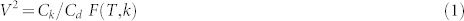
and depends on the ratio of the enthalpy coefficient (*C_k_*) to the drag coefficient (*C_d_*). These coefficients in general depend on the state of the air-sea interface changing with storm intensity. Here, *V* is the maximum surface wind speed interpreted here as a 10-min average at 10-m height, 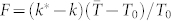 is a function of *k*, the enthalpy, k*, the surface saturation enthalpy, 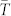, the pre-cyclone depth-averaged ocean temperature, and *T*_0_, the temperature of the outflow at the top of the tropical cyclone. The expression for thermodynamic efficiency *F*, which has an outflow temperature *T*_0_ in the denominator, is based on an assumption that all of the dissipative heating occurs in the atmospheric boundary layer^7^. The actual intensity of a storm moving quickly from a region of higher to lower potential intensity can exceed the theoretical potential intensity for its location, because some time is required for a storm to adjust to its new environment (or due to other possible limitations of the potential intensity theory itself^7^).

Laboratory experiments^8^, in part supported by field data^9^, suggest that *C_k_* may not have substantial dependence on wind speed. The *C_d_* dependence on wind speed under tropical cyclone conditions is thus critically important for understanding and modeling storm intensity. Another laboratory experiment^10^ concluded that *C_d_* increases with wind speed but levels off above approximately 33 ms^−1^ wind speed, corresponding to the transition to a Category 1 hurricane. Furthermore, according to available field data^11^,^12^,^13^,^14^, in tropical cyclones *C_d_* may even drop.

These observations indicate that the regime of air-sea interaction dramatically changes under tropical cyclone wind speeds. However, with the currently widely used sea spray generation function^15^, the leveling off or decrease of *C_d_* in tropical cyclones cannot be completely explained by the suppression of near-surface turbulence by buoyancy forces due to spray loading in the hurricane boundary layer. The spray buoyancy effect on *C_d_* appears to be relatively small^16^,^17^ when referred to 10 m height (denoted here as *C*_10_), though it may be more pronounced at greater heights^18^.

We bring forth the concept that under very high wind speed conditions, extensive generation of sea spray and foam produces a two-phase environment that suppresses short gravity-capillary waves, affecting the aerodynamic drag of the sea surface.

Under tropical cyclones, the air-sea interface is covered by the two-phase environment (“white out”^19^), which is much more widespread than whitecaps produced by typical breaking waves. According to recent analysis^19^, whitecap coverage may not exceed 10% of the surface area even in tropical cyclone conditions. The factors contributing to the white out of the sea surface outside of whitecaps are large spray droplets (spume) and smaller spray droplets, produced by the background air-bubble population.

## Results

**Figure 1:**
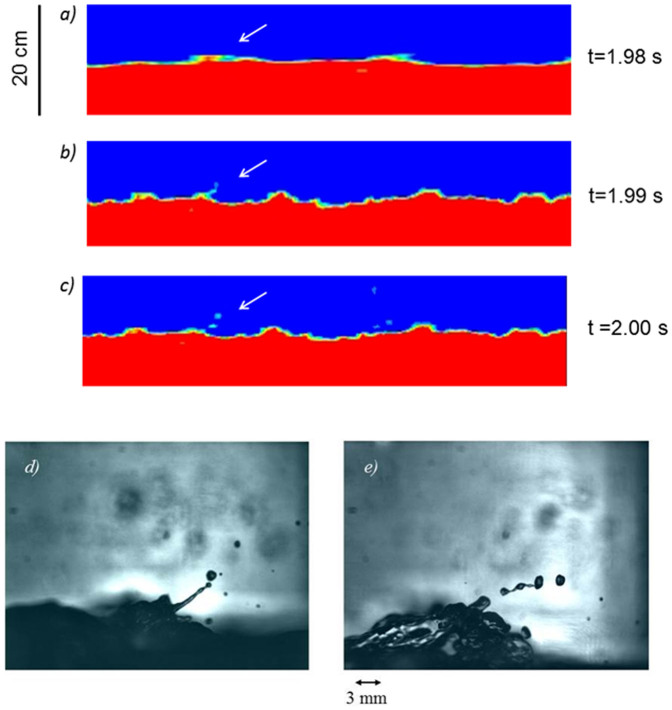
Formation of spray droplets under very high wind-speed conditions reproduced in a (a)-(c) Volume of Fluid Large Eddy Simulation (VOF LES) and (d)-(e) at the air-saltwater interface in a laboratory experiment at the UM RSMAS ASIST facility. The final stage of the KH instability at the interface of fluids with very large density difference, like water and air, typically takes place in the form of projectiles. The VOF 3D LES model^27^ included realistic sea surface tension and was forced with wind stress corresponding to *U*_10_ ≈ 40 ms^-1^. The images are taken with a shadow-imager in a small air-sea interaction tank at ASIST at wind speed corresponding to *U*_10_ ≈ 40 ms^−1^ as well.

The formation of spume at the air-sea interface can be initiated through the Kelvin-Helmholtz (KH) shear instability (Supplementary Information, Section 1). KH waves are not able to disrupt the interface under moderate winds due to stabilizing gravity and surface tension forces. Microscale wave breaking occurs but does not disrupt the interface very much. Under strong winds the growing KH waves are able to overcome gravity and surface tension forces^20^,^21^ resulting in direct disruptions of the air-sea interface and subsequent formation of large droplets–spume^22^. The final stage of the KH instability of the interface between fluids with very large density difference, such as water and air, typically organizes in the form of projectiles^22^. We reproduced this type of instability in a laboratory experiment at the UM RSMAS Air-Sea Interaction Salt Water Tank (Fig. 1a), coordinated with a Volume of Fluid Large Eddy Simulation (Fig. 1b).

The shadow-imaging technique used however cannot resolve air-bubbles because they are located inside the fluid. Dynamics of air-bubbles under tropical cyclone force winds have been studied in laboratory conditions with high-speed photography^23^. The background air-bubble population contributes in the two-phase environment at the air-sea interface but produces only relatively small spray particles, which are within a few tens of micrometers in diameter^24^. While the bubble-generated particles may significantly contribute to the marine aerosol production, the air-sea enthalpy and momentum fluxes are dominated by spume^25^.

Tollmien-Schlichting (TS) instability is another possible mechanism of direct disruption of the air-sea interface, which can take place within the viscous sublayers on either the water- or air-side of the interface. Significantly, the density ratio and viscosity ratio of the air and water are close to the critical values where either of these two instabilities, KH or TS, can take place^26^.

A long-accepted theoretical analysis^28^ showed that in the presence of wind waves the KH instability cannot develop at the air water interface under the time-averaged wind velocity profile, though it can develop over a flat interface. Laboratory^22^ and numerical^27^ experiments, conducted with monochromatic waves, unexpectedly demonstrated that KH instability of the air-water interface does take place, though predominantly near wave crests. The local conditions near the wave crest are more favorable for KH instability development because the instantaneous interfacial shear near wave crests is higher than the time-averaged shear. The characteristic time scale of the KH instability is much shorter than the periods of energy containing wind waves^29^; as a result, the KH instability develops within a relatively short time period and locally disrupts the interface. In the more general case of the turbulent atmospheric boundary layer above the wavy sea surface, wind gusts interacting with the waves result in stochastic shear intensifications triggering local KH instabilities at the air-sea interface^30^. Furthermore, stochastic gustiness-induced wave growth has been interpreted^30^ as a generalized KH instability problem. Stochastic forcing enters multiplicatively in this theory, producing exponential growth, thus extending the Miles^27^ theory for wind-wave growth as wind and turbulence level increase.

Theoretical analysis^30^ suggests that the stochastic parametric KH instability mechanism for the growth of surface water waves is sustained in a gusty turbulent flow above the random sea surface independently of the Miles wave generation mechanism. We therefore initially treat the wave-form stress and two-phase layer stress as independent entities. These stresses are then merged in a unified model.

To smoothly connect the two-phase regime (Supplementary Information, Section 1) with the well-known “Charnock” regime where wave-form induced turbulent drag is most important (Supplementary Information, Section 2), we have developed the approach (see Methods) relying on the idea that the two-phase layer cannot support surface gravity-capillary waves whose wavelengths are shorter than or comparable to its thickness because this layer is not incompressible, and because the surface is not defined on these scales. Classic surface wave theory does not (for good reasons) take these deviations from an ideal fluid interface into account, and thus we parameterize the effects of the two-phase mixture on the gravity-capillary wave spectrum. As wind speed increases, the thickness of the two-phase layer increases^27^, eliminating successively longer waves in the high wavenumber range of the wave spectrum with consequent diminishment of the air-sea drag coefficient. Because the bulk of the kinetic energy of surface waves is located within one-half wavelength (*λ*) of the surface, we assume that short gravity-capillary waves cannot be supported by the air-wave interface for *λ*/2 < *H*, where *H* is the thickness of the two-phase transition layer. The thickness of this layer (Supplementary Information, Section 1) is consistent with the skin depth of foam from 0.2 cm to 10 cm derived from passive microwave remote sensing of the sea surface in tropical cyclones^31^. In tropical cyclones, the shorter components of the wave spectrum (the so-called high-frequency tail) are also affected by rainfall^32^ and near-surface currents^33^.

**Figure 2:**
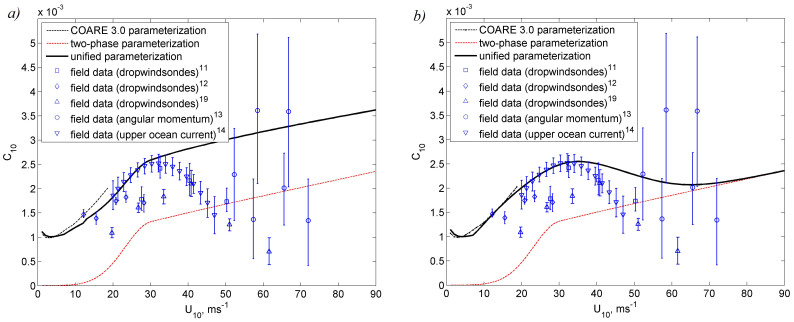
Comparison of the unified air-sea drag coefficient parameterization calculated with the surface stress method (a) and the surface roughness method (b). The COARE 3.0 parameterization, two-phase parameterization (lower bound on drag coefficient), and available data from field experiments are shown for comparison. We have included only the available field observations that report confidence intervals. The surface stress method and the surface roughness method are different approaches for unifying two-phase, wave-form, and viscous stresses (see Methods). The COARE 3.0 parameterization has been used for verification of the unified parameterizations in the range of wind speeds from 1 to 19 ms^−1^.

Unfortunately, calculations of wave-form stress with existing models of wind-wave interaction have an order of magnitude uncertainty. In operational wave models, this uncertainty is customarily compensated by introducing empirical coefficients, which are determined from field and laboratory experiments. It is, however, not clear how representative these models are under extreme wind speed conditions. Our calculations of the wave-form stress are based on two different types of wind-wave interaction models. Finally, the unified drag coefficient parameterizations (Fig. 2a, b) are calculated by either adding surface stresses^34^ or surface roughness length scales^35^ (see Methods). No consideration has been given to directional wind-wave properties, though our model could be extended to include such capability.

## Discussion

The form of the unified parameterization reflects the fundamental change of the air-sea interface properties in tropical cyclone conditions discussed earlier. We explain this change as due to the progressively stronger effects of direct disruption of the air-sea interface by KH instability and intense production of sea spray and air bubbles. In our model, the two-phase environment developing at the air sea interface eliminates some high-frequency waves, which affects the wave-form drag. Above 60 ms^−1^ wind speed, the increasing aerodynamic drag induced by the two-phase layer appreciably contributes into the unified parameterization derived with the method shown in Figure 2b. As a result, a local minimum of the drag coefficient occurs near 60 ms^−1^ wind speed.

Both versions of the unified parameterization developed here (Fig. 2) are able to explain the leveling off of the drag coefficient under hurricane wind speeds. The drag coefficient for both versions increases with wind speed until approximately 30 ms^−1^. For stronger winds, the drag coefficient either nearly levels off or even drops and increases again above approximately 60 ms^−1^. Remarkably, in one of the two methods there is a local minimum of the drag coefficient near 60 ms^−1^. (We note that the corresponding local wind stress minimum is less pronounced; however, according to eq. (1), the potential intensity depends on the drag coefficient rather than wind stress.) Scarcity of field observations under tropical cyclone conditions do not allow us to distinguish with confidence which method is preferred.

In Figure 2, it is difficult to establish a statistically significant relationship between the air-sea interface model and field data on the drag coefficient. Model verification requires expansion of observations similar to those described in ref. 11–14, 19, as well as further development of experimental techniques for extreme wind speed conditions.

Notably, the unified parameterization shown in Figure 2b has a local minimum of the drag coefficient near 60 ms^−1^ wind speed. We have investigated potential consequences of this feature on tropical cyclone dynamics.

**Figure 3:**
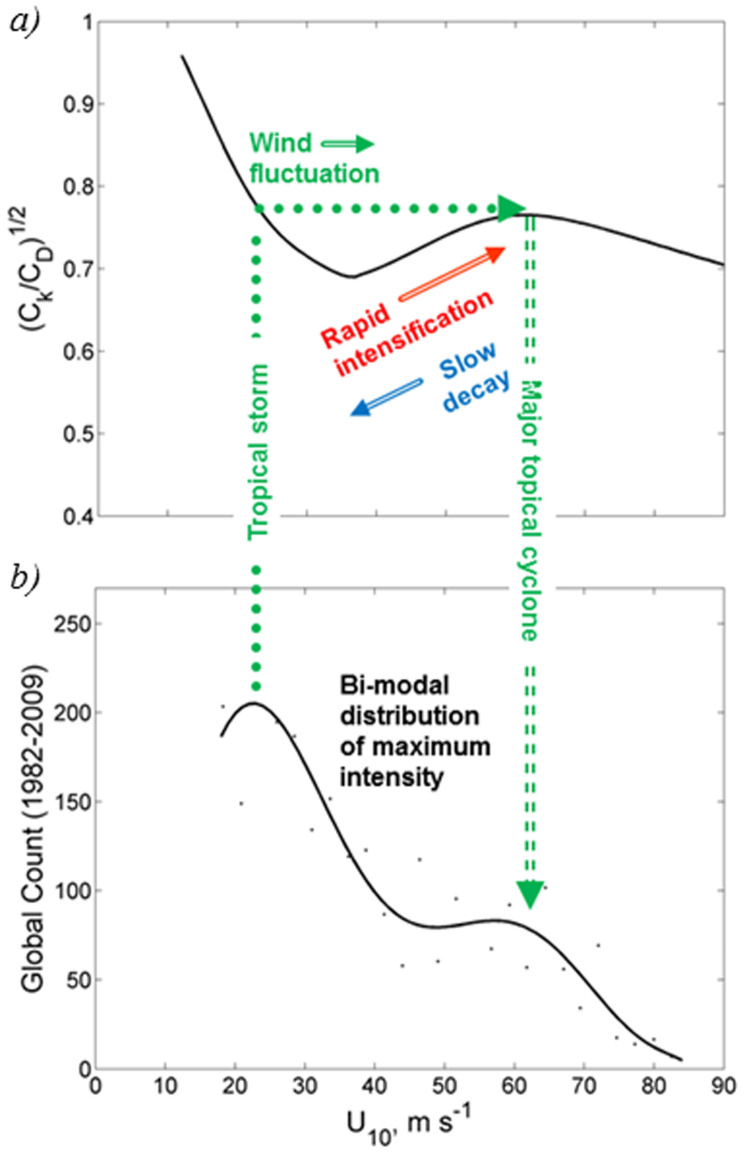
A mechanism of rapid storm intensification and the bimodal distribution of lifetime maximum tropical cyclone intensity. The shape of *C_k_*/*C_d_* dependence on wind speed containing a secondary maximum around 60 ms^−1^ (*a*) may be a factor in rapid intensification of some storms to major tropical cyclones and may explain the observed bimodal distribution of lifetime maximum intensity of tropical cyclones (*b*). Drag coefficient *C_d_* is shown in Figure 2b; while, enthalpy coefficient *C_k_* is interpolated from laboratory data^8^ for winds below 40 ms^−1^ and extended with a constant value *C_k_* = 1.2 × 10^−3^ for winds above 40 ms^−1^, which is consistent with the available field data^13^. Continuous line in (*b*) is a 7th order polynomial fit to the global best-track tropical cyclone data^38^ on maximum intensity for 1982–2009 (dots).

According to equation (1), the shape of *C_k_*/*C_d_* as a function of wind speed should have consequences for maximum tropical cyclone intensity^36^, since the potential intensity is proportional to (*C_k_*/*C_d_*)^1/2^. Under assumption of nearly constant enthalpy exchange coefficient^8^,^13^, the minimum around 60 m s^−1^ on the *C_d_* wind speed dependence (Fig. 2b) corresponds to a peak on the (*C_k_*/*C_d_*)^1/2^ = 0.75 (Fig. 3a). The positive slope of the (*C_k_*/*C_d_*)^1/2^ wind speed dependence from approximately 40 ms^−1^ to 60 ms^−1^ would introduce asymmetry in the process of storm intensification relative to storm decline in this wind speed range. Actually, observations suggest^37^ that the average hurricane-strength storm intensity declines “…at a rate roughly two-thirds that of its prior intensification”.

The positive slope of the (*C_k_*/*C_d_*)^1/2^ wind speed dependence from approximately 40 m s^−1^ to 60 m s^−1^ can thus be favorable for the rapid intensification of some storms to major tropical cyclones. However, substantial wind speed fluctuation is required to overcome the negative slope of (*C_k_*/*C_d_*)^1/2^ between approximately 30 and 40 ms^−1^ in order to initiate tropical storm intensification to a major tropical cyclone. Consequently, only a subset of tropical storms is able to overcome this barrier.

Analysis^37^ of the best track datasets obtained in the North Atlantic and western North Pacific under conditions of non-declining potential intensity also suggests that “…a given storm is equally likely to attain any intensity between hurricane force and its potential intensity.” Respectively, there is a higher probability of more intense storms at the larger value of (*C_k_*/*C_d_*)^1/2^, which may explain the observed^38^ secondary peak of lifetime maximum intensity statistics near 60 ms^−1^ resulting in a bimodal distribution (Fig. 3b). The decrease of (*C_k_*/*C_d_*)^1/2^ with wind above 60 ms^−1^ (Fig. 3a) may explain a relatively small number of storms reaching Category 5 strength (Fig. 3b).

**Figure 4:**
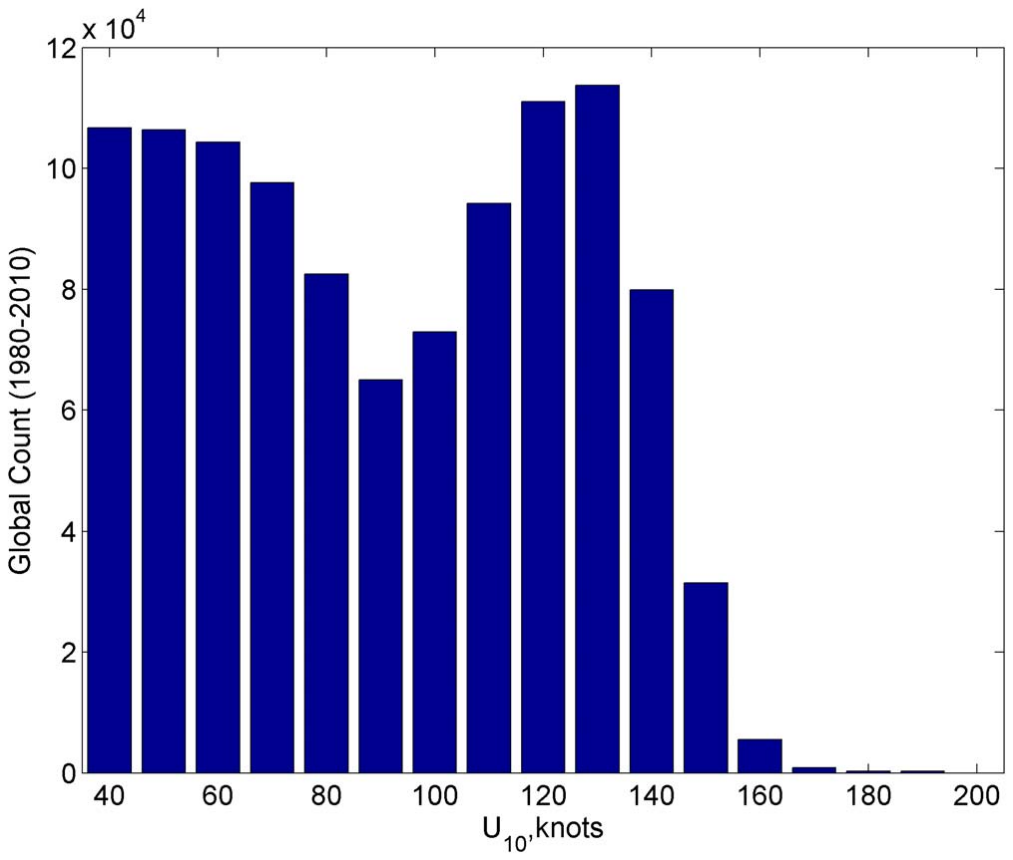
Global histogram of potential intensities calculated from NCAR/NCEP reanalyses, 1980–2010. (Courtesy of Kerry Emanuel.)

Remarkably, the potential intensity statistics derived from convective available potential energy^42^ (CAPE), calculated from NCAR/NCEP reanalysis for 1980-2010, reveal a bimodal global distribution as well (Fig. 4) (Prof. Kerry Emanuel, personal communication). The bimodal distribution of maximum intensity of tropical cyclones reported in ref. 38 can therefore be explained by environmental parameters alone, without direct involvement of *C_k_*/*C_d_* dependence on wind speed.

It should be noted that the drag coefficient estimates from upper ocean current observations^14^ are below both theoretical curves in Figure 2a, b for *U*_10_ > 40 ms^-1^, which in part could be explained by the fact that the stresses delivered to the ocean currents are less than the wind stress due to wave radiation down fetch. However, if the effect of wave stress divergence is small, then the drag coefficient estimate near 60 ms^−1^ based on the upper ocean current observations^14^ might be even lower than that predicted by the unified parameterization shown in Figure 2b. The *C_d_* estimates from dropwindsondes are also lower than the unified parameterization curve in Figure 2b. Respectively, the peak of (*C_k_*/*C_d_*)^1/2^ near 60 ms^−1^ in reality could be larger than that shown in Figure 2b and closer to unity.

Implementation of the new parameterization of the drag coefficient in operational models is expected to improve predictions of tropical cyclone intensity, storm surge, and the associated wave field.

## Methods

In order to unify the viscous, wave-form and two-phase stress regimes (see Supplementary Information for details), two approaches have been implemented. The first approach involves unification via additive stresses; while, the second approach involves unification via their impacts on the RMS surface roughness length scale.

### Surface stress method

In this approach, the exponential growth rate of the wave entering eq. (S5, Supplementary Information) is defined as follows^39^,^40^: 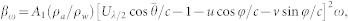
where *U*_λ/2_ is the wind speed at a height above mean water level of one half the wavelength of the growing wind wave (which is calculated assuming a logarithmic boundary layer wind profile), *u* and *v* are the near-surface current velocity components, and *A*_1_ (~0.17) is the sheltering coefficient. In this study, we do not consider currents and assume that *u* = 0 and *v* = 0. However, we note that mean currents can be strong in tropical cyclones^33^ and wave orbital motions of long waves may be significant for shorter waves.

The viscous and wave-form stresses are added as in ref. 34, while the two-phase stress is added via a gustiness formulation (S4, Supplementary Information). The three stress components, wave-form, viscous and two-phase stresses, are then added and the overall drag coefficient is computed as follows: 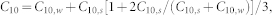
where 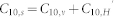.

### Surface roughness method

In this method, we adopt the exponential growth rate of the wave in response to the wind in the following form^41^:


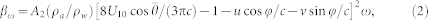


which emphases high-frequency range of surface wave spectrum in the momentum flux equation (S5, Supplementary Information), where sheltering coefficient *A*_2_ = 0.075 is determined by fitting (2) to the traditional, COARE 3.0 parameterization for *C*_10_ within the validity interval for the traditional parameterization (*U*_10_ from 1 ms^−1^ to 19 ms^−1^).

Following the approach developed in ref. 34, the wave form and viscous stresses are added as follows: 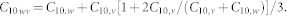


However, the two-phase drag is added via the R.M.S surface roughness length scale in the following way: 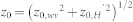.

Some justification for this method comes from the Farrell's and Ioannou conclusion^30^ that the stochastic parametric KH instability mechanism sustained in a gusty turbulent flow above the random sea surface is statistically independently of the Miles^28^ wave generation mechanism. Here, 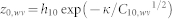, 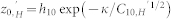. The unified drag coefficient is then as follows:
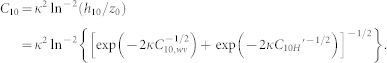


## Author Contributions

R.L. and A.S. conceived the new concept of the air-sea interface in tropical cyclones. Laboratory experiments were conducted by B.H., M.D. contributed in the air-sea interaction analysis and I.G. into tropical cyclone aspects of this work.

## Supplementary Material

Supplementary Information The air-sea interface and surface stress under tropical cyclones: Supplementary information
