# Supplementary material for: The air-sea interface and surface stress under tropical cyclones
Source: Sci Rep. 2014 Jun 16;4:5306. doi: 10.1038/srep05306 (PMC4058872; doi:10.1038/srep05306)
Supplement: Supplementary Information — The air-sea interface and surface stress under tropical cyclones: Supplementary information [file srep05306-s1.doc]

# The air-sea interface and surface stress under tropical cyclones: Supplementary information

**Alexander Soloviev^1, 2^, Roger Lukas^3^, Mark Donelan^2^, Brian Haus^2^, and Isaac Ginis^4^**

1. **KH instability at the air-sea interface and two-phase transition layer**

The acceleration of the air stream above a short KH wave induces a pressure drop^1^:

$\Delta P=P^{+}-P^{-}=\rho_{a}U^{2}kL$. (S1)

The pressure drop breaks up the interface if Δ*P* exceeds the combined restoring force of gravity and surface tension:

$\Delta P>\left(g\rho_{w}+\sigma_{s}k^{2}\right)L$. (S2)

Here: σ_s_ is the surface tension, $\rho_{w}$is the water density, $\rho_{a}$ is the air density, *k* is the wavenumber, *g* is the acceleration due to gravity, and length scale *L* is proportional to the wave amplitude. The interface breakup can occur under very high wind speed conditions^2, 3^.

The KH mechanism is possible not only at a flat air-water interface but also in the more general case of the turbulent atmospheric boundary layer above the wavy sea surface. The oceanic atmospheric boundary layer is turbulent, and wind gusts interact with the waves. A stochastic parametric KH instability can develop at the air-sea interface in the presence of wind waves even when it is not possible for the averaged air-flow conditions.

Direct disruption of the air-sea interface is a more effective mechanism for generation of the two-phase environment than large-scale wave breaking and whitecap production. Whitecap coverage is limited by only several percent even in hurricanes^4^.

Stress due to the surface roughness introduced by two-phase transition layer is calculated as follows: $\tau_{H}=\rho_{a}C_{10,H}U_{10}^{2}$ where the drag coefficient$C_{10,H}$ is found from a transcendental equation^S3^:

$C_{10,H}=\kappa^{2}\ln^{-2} \left\{1+h_{10}\left(2 m Ri_{cr}c_{z}\rho_{a}\rho_{w}\left(\rho_{w}^{2}-\rho_{a}^{2}\right)^{-1}g^{-1}C_{10,H}U_{10}^{2}\kappa^{-2}\ln^{2}\left(1+c_{z}^{-1}\right)\right)^{-1}\right\}$,

$\kappa$* *is the von Kármán constant ($\kappa=0.4$), $Ri_{cr}=0.25$, *m* is a dimensionless proportionality coefficient ($m\approx1$), $c_{z}$ is a dimensionless coefficient connecting the surface roughness length scale and the thickness of the transition layer ($c_{z}\approx0.022$),$\rho_{a}$ is the air density, and $g$ is the acceleration due to gravity, *h_10_* = 10 m. The thickness of the two-phase transition layer is then found from the equation^3^:

$H=2mRi_{cr}C_{10}U_{10}^{2}g^{-1}\kappa^{-2}\ln^{2}\left(c_{z}^{-1}+1\right)$. (S3)

Wind gusts will exceed 30 ms^-1^ when the mean wind speed is somewhat less than 30 ms^-1^. To account for the intermittency of the two-phase stress at lower winds we apply an exponential filter to the two-phase drag coefficient (equation S12) such that it is reduced by a factor of 2 at 21 m/s and is 6% at 12 m/s:

$C_{10,H}^{'}=\left\{\begin{matrix} C_{10,H}\exp[-0.0086(U_{10}-30)^{2}], & U_{10}<30ms^{-1} \\ C_{10,H}, & U_{10}\geq30ms^{-1} \end{matrix}\right.$ (S4)

1. **Wave-form stress in the two-phase environment**

The wave-form induced stress is calculated from the following formula^5^:

$\tau_{w}=\rho_{w}\int_{0}^{\infty} \int_{-\pi/2}^{\pi/2} \beta_{\omega}\omega F\left(U_{10},k,\phi\right)\cos\left(\phi\right)k d\phi dk$, (S5)

where $\rho_{w}$ is the water density, is the two-dimensional surface wave elevation wavenumber spectrum accounting for the presence of the two-phase environment (shown in Fig. S2a), $U_{10}$ is the wind speed at the standard reference height of 10 m, (*k*, *ϕ*) are polar coordinates in wavenumber space ($\phi=0$ corresponds to the local wind direction, which is also the direction of the wind stress because the wind in classic wave theory is assumed to be steady and uniform), *ω* is the angular frequency, $\beta_{\omega}=\left(1 /F\right) \partial F /\partial t$ is the exponential growth rate of the spectral components in response to the wind. We consider only the downwind semicircle, for the sake of simplicity assuming that the contribution to the wave-induced stress from waves propagating against the wind is relatively small. The wave-form drag coefficient is defined as follows: $C_{10,w}=\tau_{w}/(U_{10}^{2}\rho_{a})$.

The directional wave elevation spectrum is represented in the following way^6, 7^:

$F\left(k,\phi\right)=\left(2\pi\right)^{-1}k^{-4}\left[B_{l}+B_{h}\right]\left[1+\Delta\left(k\right)\cos\left(2\phi\right)\right]$. (S6)

The longwave curvature spectrum *B_l_* in (S6) is as follows^7^:

$B_{l}=0.5 \alpha_{p}F_{p}c_{p}/c$, (S7)

where $c=\left(g/k+k\sigma_{s}/\rho_{w}\right)^{1/2}$, $\Omega=U_{10}/c_{p}$, $F_{p}=L_{PM}J_{p}\exp\left\{-\left(\Omega/\sqrt{10}\right)\left[\left(k/k_{p}\right)^{1/2}-1\right]\right\}$, $L_{PM}=\mathrm{e}^{-1.25\left(k_{p}/k\right)^{2}}$, and $J_{p}=\gamma^{\Gamma}$. The other variables are defined as follows: $\gamma=1.7$for $0.84<\gamma<1$ and $\gamma=1.7+6 \log_{10}\left(\Omega_{c}\right)$ for$1<\Omega_{c}<5$; $\Gamma=\exp\left[-\left(\sqrt{k/k_{p}}-1\right)/\left(2\sigma^{2}\right)\right]$, $\sigma=0.08\left(1+4\Omega_{c}^{-3}\right)$, $\alpha_{p}=0.006\Omega_{c}^{0.55}$, $0.84<\Omega_{c}<5$, $k_{p}=k_{0}\Omega_{c}^{2}$, $k_{0}=g/U_{10}^{2}$, *g* is the acceleration due to gravity, $\Omega_{c}=\Omega\cos\left(\bar{\theta}\right)$, $\bar{\theta}$is the angle between the wind and the dominant waves at the spectral peak, which is limited to small values^5^ and for simplicity is taken here as $\bar{\theta}=0$.

The shortwave curvature spectrum is defined as follows^6^:

$B_{h}=0.5\alpha_{m}f_{m}c_{m}/c$, (S8)

where $\alpha_{m}=\alpha_{0}u_{*}/c_{m}$ is the generalized Phillips-Kitaigorodskii equilibrium range parameter for short waves, $\alpha_{0}=1.4\times10^{-2}$, $c_{m}=\sqrt{2g/k_{m}}$=0.23 m s^-1^ is the minimum phase speed at the wavenumber *k_m_*, which is also associated with the gravity-capillary peak in the curvature spectrum. In order to account for viscous cut-off and the bandwidth of gravity-capillary waves, a roll off function^6^

$f_{m}=\mathrm{e}^{-0.25\left(k/k_{m}-1\right)^{2}}$. (S9)

was originally introduced (dashed line in Figure S1), effectively suppressing the portion of the wavenumber spectrum$k>k_{m}$. According to Figure S1, this function also suppresses by ~20% a portion of the wavenumber spectrum, $k<k_{m}$, which is an undesirable effect. In addition, it is well known that the shortwave spectrum $B_{h}$ in the original formulation^6^ is unbounded on the low wavenumber side of the spectrum.

In order to address these issues, we have developed the following formulation for the short wave roll off function:

$f_{m}=e^{-0.0125\left(k/k_{m}\right)^{4}}\left(1-e^{k/k_{0}}\right)^{5}$, (S10)

where $k_{0}=0.1g/u_{*}^{2}$. This function is shown in Figure S1 with a family of curves calculated for the wind speed range from 5 ms^-1^ to 85 ms^-1^. Notably (S10) provides practically the same roll off as (S9) at the higher edge of the wavenumber range but does not have the 20% drop in the lower wavenumber range. As well it bounds the shortwave spectrum $B_{h}$ wavenumber from the lower edge of the wavenumber range. Parametric dependence of $k_{0}$ on friction velocity $u_{*}$ also provides smooth joining of$B_{l}$ and $B_{h}$ within the large range of wind speed conditions.


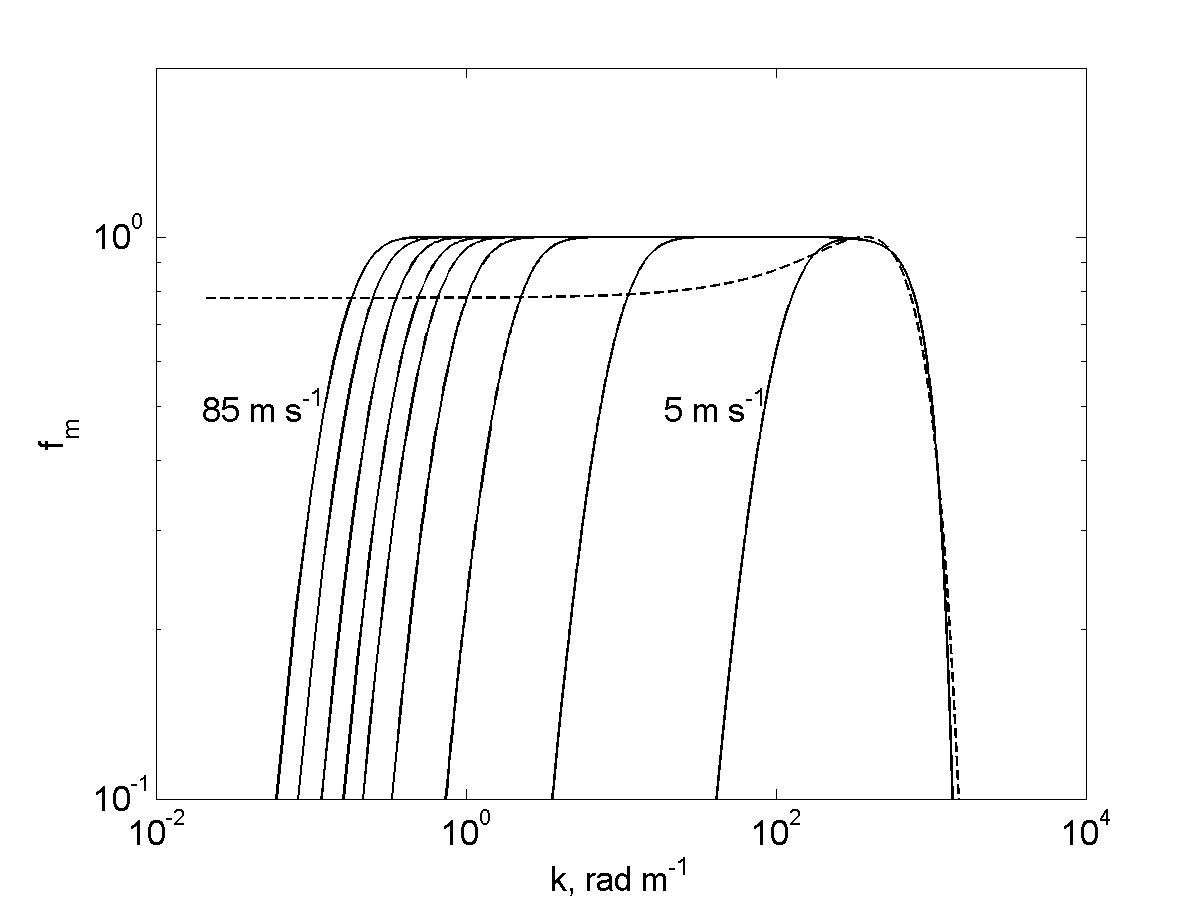


Figure S1. **Original^6^ and modified shortwave roll off function.** Function (S8) is shown by dashed line; while, the new formula (S10) represented by a family of continuous lines corresponding to the wind speed increasing from 5 ms^-1^ to 85 ms^-1^ with 10 ms^-1^ increments.

We assume that the short gravity-capillary waves cannot be supported by the air-wave interface under condition

$\lambda/2<H$, (S11)

where $H$ is the thickness of the two-phase transition layer defined from equation (S3). Equation (S11) is equivalent to the condition$k>k_{cutoff}$, where

$k_{cutoff}= \pi/H$. (S12)

In order to take into account the effect of the two-phase environment suppressing shorter waves, we replace $k_{m}$ in (S9) with$k_{mw}$defined by formula$k_{mw}=\left(k_{m}^{-4}+k_{cut}^{-4}\right)^{-1/4}$, which produces smooth transition from the viscous to two-phase layer cut off wavenumber .

Figure S2a shows the model surface elevation saturation spectra taking into account presence of a transition layer. Figure S2b shows the anticipated effect of two phase transition layer on the curvature saturation spectrum, which characterizes “sharpness” of waves and, thus, aerodynamic properties of the sea surface.


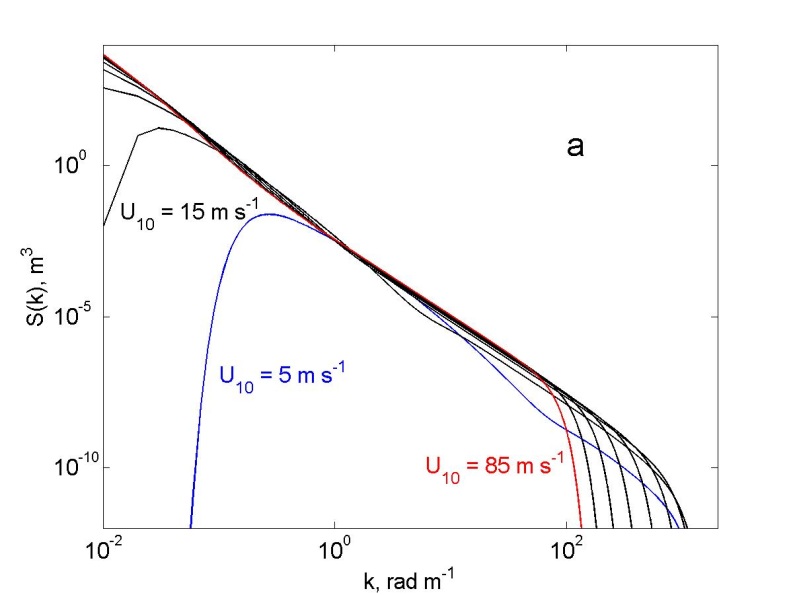

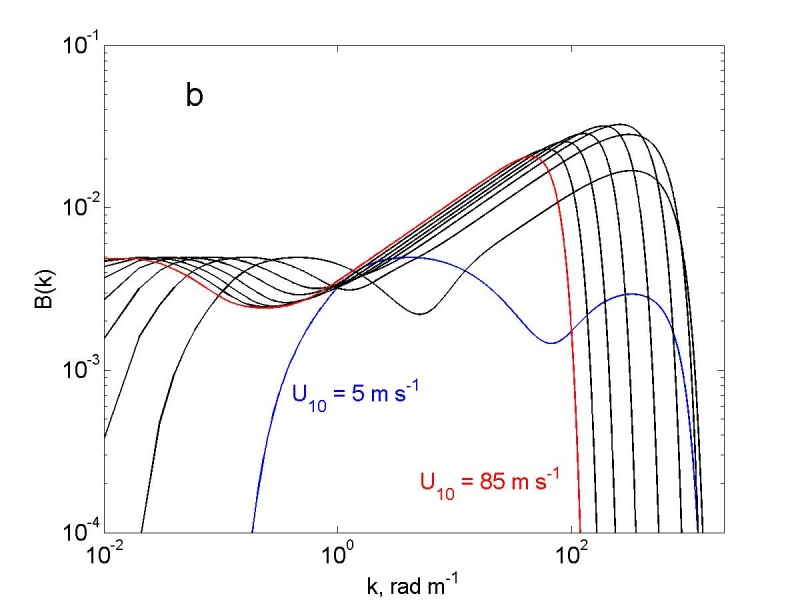


**Figure S2 | Wave surface elevation (a) and curvature (b) saturation spectra taking into account the suppression of short waves by the two-phase environment under a range of wind speeds including hurricane conditions.** Wind speed at 10 m height ($U_{10}$) increases from 5 ms^-1^ to 85 ms^-1^ with 10 ms^-1^ increments.

Experimental and theoretical results^8^ reveal the tendency of the short wave spectrum to saturate under high wind speed conditions, which has been incorporated in the model spectrum^6^ that we use here. Direct measurement of surface gravity-capillary waves is extremely challenging under tropical cyclone conditions. Indirect data from microwave radar scattering studies appear to be somewhat helpful in verification of the wave models in the gravity-capillary range^7,9,10^. Laboratory experiments are also a valuable source of information on the dynamics of gravity-capillary waves^11,12^, though limited by the dimensions of the laboratory tank. Passive acoustic remote sensing is another potentially important source of information on the properties of the gravity-capillary range of surface waves in real ocean conditions, especially for wave directional properties^13^.

1. **Viscous stress**

Viscous stress is calculated in the following way: $\tau_{\nu}=\rho_{a} C_{10,\nu} U_{10}^{2}$, where the viscous drag coefficient $C_{10,\nu}$ is parameterized via the surface roughness length scale $z_{0,\nu}=0.11 \nu_{a}/u_{*a}$ using the classic logarithmic wind profile assumption: $C_{10,\nu}=\kappa^{2}/\ln^{2}\left(h_{10}/z_{0,v}\right)$, where $\nu_{a}$ and $u_{*a}$ are the molecular viscosity and friction velocity in the air, respectively.

**References to Supplementary Information**

1. Hoepffner J., Blumenthal, R. & Zaleski, S. Self-similar wave produced by local perturbation of the Kelvin-Helmholtz shear-layer instability. *Phys. Rev. Lett.* **106**, 104502-1 - 104502-4 (2011).
2. Koga, M. Direct production of droplets from breaking wind-waves-Its observation by a multi-colored overlapping exposure technique. *Tellus* **33,** 552-563 (1981).
3. Soloviev, A., Fujimura, A. and S. Matt. Air-Sea Interface in Hurricane Conditions*. J. Geophys. Res.* **117,** C00J34 (2012).
4. Holthuijsen, L. H., Powell, M.D. & Pietrzak, D. Wind and waves in extreme hurricanes. *J. Geophys. Res.* **117**, C09003 (2012).
5. Caudal, G. Self-consistency between wind stress, wave spectrum, and wind-induced wave growth for fully rough air-sea interface*. J. Geophys. Res.* **98**, 22,743-22,752 (1993)
6. Elfouhaily, T., Chapron, B., Katsaros, K., and Vandemark, D. A unified directional spectrum for long and short wind-driven waves. *J. Geophys. Res.* **102**, 15,781–15,796 (1997).
7. Donelan, M. A. & Pierson Jr., W.J. Radar scattering and equilibrium ranges in wind-generated waves with application to scatterometry*. J. Geophys. Res*. **92**, 4971– 5029 (1987).
8. Hwang, P.A., Burrage, D.M., Wang, D.W. & Wesson, J.C. Ocean Surface Roughness Spectrum in High Wind Condition for Microwave Backscatter and Emission Computations. *J. Atmos. Oceanic Technol.* **30**, 2168–2188 (2013).
9. Bjerkaas, A. W. & Reidel, F.W. Proposed model for the elevation spectrum of a wind-roughened sea surface. Rep. APL-TG-1328-1-31, 31 pp., Appl. Phys. Lab., Johns Hopkins Univ., Laurel, Md (1979).
10. Apel, J. R. An improved model of the ocean surface wave vector spectrum and its effects on radar backscatter. *J. Geophys. Res*. **99,** 16,269–16,291 (1994).
11. Jähne, B. and K. S. Riemer, K.S. Two-dimensional wave number spectra of small-scale water surface waves. *J. Geophys. Res.* **95,** 11,531–11,546 (1990).
12. Hara, T., Bock, E.J. & Lyzenga, D. In situ measurements of capillary-gravity wave spectra using a scanning laser slope gauge and microwave radars. *J. Geophys. Res.* **99,** 12,593–12,602 (1994).
13. Ardhuin, F., J.-A. Royer, B.M. Howe, R. Lukas, J. Aucan. A numerical model for ocean ultra low frequency (ULF) noise: wave-generated acoustic-gravity and Rayleigh modes. *J. Acoust. Soc. Am.* **134**, 3242-59 (2013).
